# Similarity of Body Size in Queens of the Wood ant *Formica aquilonia* from Optimal and Sub-Optimal Habitats Indicates a Strong Heritable Component

**DOI:** 10.1673/031.013.11501

**Published:** 2013-10-26

**Authors:** Marja-Katariina Haatanen, Jouni Sorvari

**Affiliations:** 1 Department of Biology, Section of Ecology, FI-20014 University of Turku, Finland; 2 Department of Environmental Science, University of Eastern Finland, P.O. Box 1627, FI-70211 Kuopio, Finland

**Keywords:** forest clear-cutting, habitat quality, phenotype, red wood ants

## Abstract

Body size in animals is affected by both genes and the environment (e.g., the amount of food resources). In ants, body size is related to several traits in an individual's physiology and life history. For example, a large queen may increase offspring production, thus increasing her overall fitness. In this study, whether sub-optimal environmental conditions affect the body size of queens of the red wood ant, *Formica aquilonia* Yarrow (Hymenoptera: Formicidae). The sizes (head width in mm) of virgin queens, i.e., gynes, originating from forest interiors (resource rich) and from commercial forest clear-cuts (resource poor) were measured. No differences in the body size of the queens from the two habitats were found. In addition, the within-nest variation in queen size was similar between habitat types. The results indicate that the body size variation of *F. aquilonia* queens is not sensitive to environmental variation, unlike *F. aquilonia* workers. The lack of environmental variation in queen size in *F. aquilonia* may be due to a strong selection in the past to monomorphic size in this obligately polygynous (multi-queened) species.

## Introduction

The phenotype of an organism is a consequence of both the genes it inherits and the environment in which those genes are expressed. Although the genotype determines the developmental limits of an individual, an individual's phenotype is usually also the result of many environmental influences (Metcalfe and Monanghan 2001). For example, food availability is a very important environmental factor affecting the growth of an individual animal (Zanette et al. 2000; Hemmi and Jormalainen 2002).

Social insects provide a challenge and an opportunity to examine the effects of environmental and genetic factors on the phenotype of an individual. Social insects and their body size variation are particularly interesting because of their complex social organization, which includes at least 2 morphologically different female castes, i.e., workers and queens (Wilson 1971; Kovacs et al. 2009). It is very common, particularly in ants and termites, for workers to be sizepolymorphic, or their size distribution to be unimodal yet highly variable (Wilson 1971). In addition, several ant species have queens that are highly variable or dimorphic in size (e.g., Howard 2006; Rosset and Chapuisat 2007).

Large body size makes a difference in the life of a social insect. Among queens, large body size has been associated with better survival, better immune defense, and higher reproductive success (Beekman et al. 1998; Wiernasz and Cole 2003; Fjerdingstad and Keller 2004; Sorvari et al. 2008). In addition, large male ants may have better mating success than smaller ones (Abell et al. 1999; Wiernasz et al. 2001). Previous studies have shown that body size in ant workers is associated with survival in harsh conditions (Lighton et al. 1994; Fournier et al. 2007; Clémencet et al.2010).

While adult body size in ants is often strongly influenced by environmental factors, genes are also involved. The size of ant workers in *Formica truncorum*, *F. podzolica*, and *Cataglyphis cursor* appears to be largely environmentally determined (Deslippe and Savolainen 1995; Bargum et al. 2004; Fournier et al. 2007). Conversely, it has been found that body size in the workers of *Leptothorax acervorum* is to some extent genetically determined, although it may follow different reaction norms in different populations (Heinze et al. 2003). Similarly, there was a strong maternal effect on the body size of workers of the acorn ant, *Temnothorax curvispinosus* (Linksvayer 2006). In addition, whether workers develop into minor or major workers seems to be linked with genes in the Australian sugar ant, *Camponotus consobrinus* (Fraser et al. 2000), and likely also in other ants with polymorphic workers.

Body size in queens also seems to be strongly genetically determined in some ant species, but more environmental in some other species. For example, indicating stronger genetic determination, the size of *Formica podzolica* queens was similar between high and low quality habitats (Deslippe and Savolainen 1994), and there seemed to be a strong maternal effect in the size of queens in the queensize dimorphic species *Temnothorax rugatulus* (Rüppell et al. 2001a) and the monomorphic *T. curvispinosus* (Linksvayer 2006). Further, significant heritability for *F. truncorum* queen size was found in one study year, but not in another, indicating that under certain circumstances environmental factors may override the influence of genes (Bargum et al. 2004). Concurrent genetic and environ mental effects on queen size variation have also been found in the ant *Lasius niger* (Fjerdingstad 2005). Finally, it was found that a protein-rich diet increased the size of the queens, workers, and males produced in the ant *Linepithema humile* (Aron et al. 2001). Therefore, it seems that the relative roles of environment and genes on body size determination may depend on both the species and caste.

Although environment does not always seem to be the stronger determining factor in regulating the body size of queens, it is particularly interesting to investigate whether striking differences in habitat quality, e.g., forest versus forest clear-cut, might make a difference, because body size may be linked with better survival, reproductive success, and immune defense. Forest clear-cutting is the most common forest harvesting method in Finland. This deforestation radically decreases the habitat quality of wood ants. Clear-cutting seems to cause food resource limitation for wood ants, especially for the red wood ants of *Formica rufa* group (Sorvari and Hakkarainen 2009; Sorvari et al. 2011). In addition, both the micro-climate and uppermost soil layers may become drier in clear-cut areas (Keenan and Kimmins 1993), which may alter the temperature and humidity regulation of the mound nests of wood ants (Rosengren et al.1979; Sorvari and Hakkarainen 2009). Body size in the workers of the red wood ant *Formica aquilonia* Yarrow (Hymenoptera: Formicidae) is often smaller in clear-cut areas, and the small body size has been suggested to be caused by changed biotic and abiotic conditions (Sorvari and Hakkarainen 2009). In the same study, worker size was shown to increase with nest size in forests but not in clear-cut areas. Thus, clear-cutting provides an excellent opportunity to test the effects of the environment and genes on the body size of wood ant queens.

The objective of this study was to determine whether poor environmental conditions affect the body size of queens of the wood ant *F.aquilonia*. This was examined by comparing the sizes of young, unmated, winged queens (i.e., gynes) originating from forest interiors (food rich) and commercial forest clear-cuts (food limited). Environmental factors (i.e., lack of resources) can vary within the same habitat and thus may exert different kinds of effects upon the queens. Under food limitations, some queen larvae may become poorly fed, whereas others may receive a sufficient amount of food. This may cause the withinnest variation in queen sizes to increase. Therefore, the body size variation within nests in different environments also was studied.

## Materials and Methods

### Study species and study area

*F. aquilonia* is the most common wood ant in northern European boreal forests (e.g., Punttila and Kilpeläinen 2009). It is a typical species in sparse to medium density mature forest stands and forest edges from Scotland to Siberia (Collingwood 1979; Czechowski et al. 2002). It is a highly polygynous (multiple queens) and polydomous (multiple nests) species, with large nest mounds containing over a million workers and hundreds of queens (Pamilo 1982; Rosengren et al. 1987). *F. aquilonia* has ecological effects over several trophic levels and is threatened by commercial forest clear-cutting, which reduces the production of sexual offspring, nest survival, and food resources (Sorvari and Hakkarainen 2005, 2007a, 2009) and creates a bias upon the sex ratio of sexual offspring (Sorvari and Hakkarainen 2007b).

The study population was located in the boreal coniferous zone in central Finland, near the town of Jyväskylä (62° 14′ N, 25° 44′ E). Sixteen separate (between distances > 1 km) bilberry, *Vaccinium myrtillus* L. (Ericales: Ericaceae), growth forest stands dominated by over 70 years old Norway spruce, *Picea abies* (L.) H. Karst (Pinales: Pinaceae), were used. Eight had been logged 2–3 years before the study, whereas the remaining 8 were uncut. Of the 27 study nests, 14 were located in 8 different forest patches (more than 50 m away from the forest edges), and 13 nests in 8 different clear-cut areas. Young, unmated, winged queens (gynes) were collected by hand from the nest mound interiors (mean number of queens/nest ± 95% CL, forests: 27.3 ± 8.3, clear-cuts: 24.5 ± 8.6; *F*_1, 25_ = 0.94, *p* = 0.34). The relatedness among nestmate queens is usually extremely low in *F. aquilonia* (*r* is typically close to zero; Sundström et al. 2005), thus it is very likely that the sampled queens were mostly not sisters.

### Measurements

Body size instead of body weight was measured because gynes gain weight after hatching from pupa (Keller and Passera 1989), thus body size better describes the morphometric growth of an individual. The maximum head width above the eyes has been used as a standard measurement of body size in ants of the genus *Formica* (e.g. Deslippe and Savolainen 1994; Sorvari and Hakkarainen 2009). The maximum head width of a total of 700 queens was measured using an Olympus SZ40 microscope (40 × magnification) and an Olympus 24–10/100 ocular micrometer (www.olympus-global.com), which allowed accurate measurements to the nearest 0.05 mm.

Counting all the ants in each colony would have been impractical to estimate colony sizes. The basal diameter and area of nest mounds have been shown to correlate positively with the worker population in moundbuilding *Formica* ants (Seifert 1991; Liautard et al. 2003) and other *Formica* species (Deslippe and Savolainen 1994). Thus, the basal area of nest mounds was used to estimate the colony size. However, it is not known how strongly nest size and worker population is correlated in poor environments such as clearcuts. The shape of the nest mound base of the study species varied from near-circular to ellipsoid, therefore the basal area of each nest mound was calculated (in m^2^) using the following area formula for an ellipse:

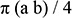
where a and b represent the maximum and minimum basal diameters measured. Since the nests were not excavated to see how deep they penetrated, the nest mound volume was not investigated.

### Statistical procedures

General linear mixed models with a Kenward-Roger approximation of degrees of freedom were used to analyze queen size difference and size variation difference. Due to multiple individuals from the same nest and 1 –5 nests from the same study stand (*F. aquilonia* forms multi-nest coalitions), the nest of origin nested within the study stand was used as a random factor in the model of head width difference between forests and clear-cuts. The effect of the random factor was tested by comparing the model with the random factor and the same model without the random factor with the likelihood ratio test (see Littell et al. 2006). In the models of nest size effect and within nest variation difference (measured as the standard deviation of head widths) between forests and clear-cuts, only the study stand was used as a random factor because each nest got only 1 value of mean and standard deviation of queen size. In the model of nest size effect, the nest mean size of queens was used because each nest got only 1 nest size. The general linear mixed models were analyzed using the MIXED procedure in SAS statistical software, version 9.3 (SAS Institute, www.sas.com). In addition, the similarity of size distributions of queens between habitat types was analyzed using a Kolmogorov- Smirnov (1 independent samples) test with PASW Statistics software, version 18.0 (SPSS Inc., IBM, www.ibm.com).

## Results

**Figure 1.**
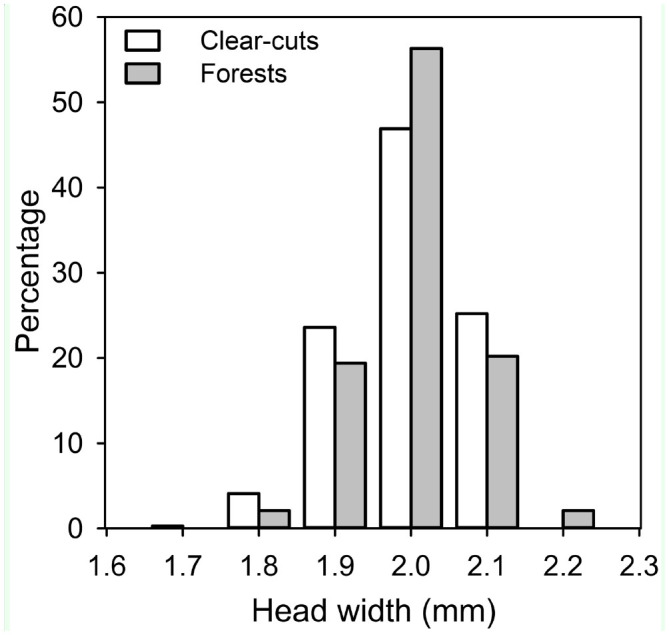
Histogram of the head widths of *Formica aquilonia* queens originating from forest, as well as clear-cut areas. The size distribution is similar. Number of gynes and nests (respectively) in clear-cuts is 318 and 13, and in forests 382 and 14. High quality figures are available online.

The queen size, measured as head width, variedfrom 1.71 to 2.18 mm and from 1.81 to 2.24 mm in clear-cut and forest interior populations, respectively (Figure 1). Interestingly, the minimum and maximum sizes were smaller in queens from clear-cuts than those from forest interiors. However, the overall size of queens did not differ between clear-cut and forest interior populations (mixed model based least squares mean ± 95% CL: clearcuts 2.001 ± 0.03, forest interiors 2.015 ± 0.03; *F*_1, 24.1_ = 0.42, *p* = 0.52). The nest of origin nested within the study stand had a significant effect in the model (χ^2^ = 271.3, df = 1, *p* < 0.0001). The nest mound size (as an estimate of colony size) did not affect the colony mean queen size, and the relationship between nest size and queen size was similar in both habitat types (nest size: *F*_1, 20.6_ < 0.01, *p* = 0.96; interaction term habitat type X nest size: *F*_1, 18.4_ < 0.01, *p* = 0.99).

The within-nest size variation (SD) of queens did not differ between clear-cut and forest interior populations (mixed model based least squares mean ± 95% CL: clear-cuts 0.062 ± 0.01, forest interiors 0.057 ± 0.01; *F*_1, 24_ = 1.99, *p* = 0.17). The number of queens examined per nest did not affect the within-nest size variation (*F*_1, 24_ = 1.31, *p* = 0.26). Furthermore, the size distribution of queens was similar between the habitat types (Kolmogorov-Smirnov Z = 0.86, *p* = 0.45; Figure 1).

## Discussion

It was found in a previous study that while there is a strong heritable component in the size of queens of *F. truncorum* (a close relative to *F. aquilonia*), it may be overridden by environmental conditions (Bargum et al.2004). Although forest clear-cutting is a rather strong environmental change for forestdwelling wood ants like *F. aquilonia* and clearly affects the size of its workers (Sorvari and Hakkarainen 2009), for some reason it did not affect the size of queens. Thus, the size of queens seems to be less sensitive to environmental variation than the size of workers in *F. aquilonia*. In addition, the similarity between the size variations in the 2 different habitat types gives further support to the interpreta tion that queen body size is mainly determined by some heritable component in *F. aquilonia*.

Because *F. aquilonia* normally lives in polydomous colonies, the lack of size differences could be mediated due to food sharing between neighboring nests. However, an earlier study with the same colony populations showed that the neighboring nests become competitors (Sorvari and Hakkarainen 2004). In addition, the decrease in the size of workers in clear-cuts in the same study stands (Sorvari and Hakkarainen 2009) supports the suggestion that food sharing between clear-cut nests and forest nests is not working or does not support the clear-cut nests enough.

The queen size can vary between single- and multiple-queen colonies (monogyny vs. polygyny). In many cases of socially polymorphic ant species, the queens in monogynous colonies are generally larger than those in polygynous colonies (Herbers 1984; Keller and Ross 1993; McInnes and Tschinkel 1995; Hamaguchi and Kinomura 1996; Kikuchi et al. 1999; Buschinger and Schreiber 2002; Rosset and Chapuisat 2007; Meunier and Chapuisat 2009). This, however, is not a rule in all socially polymorphic ant species (see Murase et al. 2000; Rüppell et al. 2001b).

In queen size-dimorphic species, large queens (macrogynes) are capable of establishing new colonies independently, whereas small queens (microgynes) are specialized in dependent colony founding by returning to the natal colony (leading to polygyny; e.g. McInnes and Tschinkel 1995; Rüppell et al. 2001c). This may also be a general rule in most cases of ants with size-dimorphic queens. In support, it has been found that queen body size and colony founding strategy may both be influenced by the same gene (DeHeer et al. 1999).

Large body size may be advantageous in queens that establish their colonies solitarily. Although the phenomenon has yet not been studied in queens, large body size in ant workers seems to protect against both high and low temperatures (Heinze et al. 2003; Clémencet et al. 2010). Large body size may also protect queens against desiccation. In harvester ants, larger queens lose water at a significantly lower rate (Wiernasz and Cole 2003). Further, in *F. aquilonia*, larger queens normally have a better pathogen encapsulation rate than smaller individuals (Sorvari et al. 2008). However, this association was reversed in clear-cuts, suggesting that queens cannot invest concurrently in both large body size and effective immune functions in a resourcepoor environment. Production of good quality queens may be possible in clear-cuts by reducing the number of produced gynes.

*F. aquilonia* is an obligately polygynous species, i.e., it may have monogynous colonies only on very rare occasions. Queens of *F. aquilonia*, as well as the queens of the other species in a *Formica rufa* group, can establish new colonies via temporary parasitism by taking over a nest of *Formica* (*Serviformica*) sp. (Gösswald 1989; Buschinger 2009). In temporary parasitism, the parasitic species depends on a host species only during the founding phase of new colonies by young queens (Buschinger 2009). However, this is an extremely rare way to found a colony in *F. aquilonia* because a mated queen of *F. aquilonia* almost always returns to her natal or other conspecific nest and joins the existing queen pool of the colony (Rosengren et al. 1993). Therefore, the survival of a colony in different habitats is not so dependent upon the large size of the queen, which is contrary to species that found colonies independently (i.e., by a single queen).

When queens tend to join their natal colonies, and if the size is strongly mediated by heritable factors, the size of nestmate queens should be similar, and the possible variation should exist between colonies from different areas. In accordance, there was significant variation between colonies, i.e., a significant effect of the random variable nest within an area. This again may be a sign of the strong genetic component of queen size in *F. aquilonia*.

Environmental variation in worker size may not be selected against because it is beneficial for colonies to have workers of different sizes for different tasks (e.g., Billick and Carter 2007; Evison and Ratnieks 2007). By contrast, queens have less diverse tasks, therefore they may have a certain optimal size (or sizes in cases of species with di- or tri-morphic queens), which may, particularly in obligately polygynous species with a single colony founding strategy, be mostly free from environmental variation.

The results of our study did not show effects of forest clear-cutting on body size of queens, but clear-cutting has been shown to decrease production of sexual offspring (Sorvari and Hakkarainen 2005, 2007c) and change the relationship between body size and immunity in queens of *F. aquilonia* (Sorvari et al. 2008). While the body size is not affected, the number and quality of produced queens seem to be altered.
